# CRISPR-Cas12a-Empowered Electrochemical Biosensor for Rapid and Ultrasensitive Detection of SARS-CoV-2 Delta Variant

**DOI:** 10.1007/s40820-022-00888-4

**Published:** 2022-08-04

**Authors:** Chenshuo Wu, Zhi Chen, Chaozhou Li, Yabin Hao, Yuxuan Tang, Yuxuan Yuan, Luxiao Chai, Taojian Fan, Jiangtian Yu, Xiaopeng Ma, Omar A. Al-Hartomy, S. Wageh, Abdullah G. Al-Sehemi, Zhiguang Luo, Yaqing He, Jingfeng Li, Zhongjian Xie, Han Zhang

**Affiliations:** 1 International Collaborative Laboratory of 2D, Materials for Optoelectronics Science and Technology of Ministry of Education, Institute of Microscale Optoelectronics, College of Physics and Optoelectronic Engineering, Shenzhen University, Shenzhen, 518060 People’s Republic of China; 2 Shenzhen International Institute for Biomedical Research, Shenzhen, 518116 People’s Republic of China; 3 Department of Respiratory, Shenzhen Children’s Hospital, Shenzhen, 518038 People’s Republic of China; 4 Institute of Pediatrics, Shenzhen Children’s Hospital, Shenzhen, 518038 People’s Republic of China; 5 Shenzhen Han’s Tech Limited Company, Shenzhen, 518000 People’s Republic of China; 6 Hospital of Guangzhou Medical University, Qingyuan city People’s Hospital, Qingyuan, 511518 People’s Republic of China; 7 Department of Physics, Faculty of Science, King Abdulaziz University, Jeddah, 21589 Saudi Arabia; 8 Research Center for Advanced Materials Science (RCAMS), King Khalid University, P.O. Box 9004, Abha, 61413 Saudi Arabia; 9 Department of Chemistry, College of Science, King Khalid University, P.O. Box 9004, Abha, 61413 Saudi Arabia; 10 Zhongmin (Shenzhen) Intelligent Ecology Co., Ltd, Shenzhen, 518055 People’s Republic of China; 11 Shenzhen Center for Disease Control and Prevention, Shenzhen, 518055 People’s Republic of China

**Keywords:** SARS-COV-2 variant, Methodology of electrochemical CRISPR sensing (MOECS), Gold nanoparticles (AuNPs), Point-of-care testing (POCT)

## Abstract

Coronavirus disease 2019 (COVID-19) is a highly contagious disease caused by severe acute respiratory syndrome coronavirus 2 (SARS-CoV-2). The gold standard method for the diagnosis of SARS-CoV-2 depends on quantitative reverse transcription-polymerase chain reaction till now, which is time-consuming and requires expensive instrumentation, and the confirmation of variants relies on further sequencing techniques. Herein, we first proposed a robust technique-methodology of electrochemical CRISPR sensing with the advantages of rapid, highly sensitivity and specificity for the detection of SARS-CoV-2 variant. To enhance the sensing capability, gold electrodes are uniformly decorated with electro-deposited gold nanoparticles. Using DNA template identical to SARS-CoV-2 Delta spike gene sequence as model, our biosensor exhibits excellent analytical detection limit (50 fM) and high linearity (*R*^2^ = 0.987) over six orders of magnitude dynamic range from 100 fM to 10 nM without any nucleic-acid-amplification assays. The detection can be completed within 1 h with high stability and specificity which benefits from the CRISPR-Cas system. Furthermore, based on the wireless micro-electrochemical platform, the proposed biosensor reveals promising application ability in point-of-care testing.

**Supplementary Information:**

The online version contains supplementary material available at 10.1007/s40820-022-00888-4.

## Highlights

- A robust technique-methodology of electrochemical CRISPR sensing is first proposed for the rapid, highly sensitive and specific detection of SARS-CoV-2 variant without any nucleic-acid-amplification assays.
- Using the DNA template identical to SARS-CoV-2 Delta spike gene sequence as model, our biosensor exhibited excellent analytical detection limit (50 fM) and high linearity (*R*^2^ = 0.987) without any amplification assay.
- Specific crRNA was designed to match the mutation site on nucleic acid sequence of the SARS-CoV-2 Delta variant, presenting programmability, universality, and scalability for diagnosis of other emerging SARS-CoV-2 variants.

**Supplementary Information:**

The online version contains supplementary material available at 10.1007/s40820-022-00888-4.

## Graphical abstract

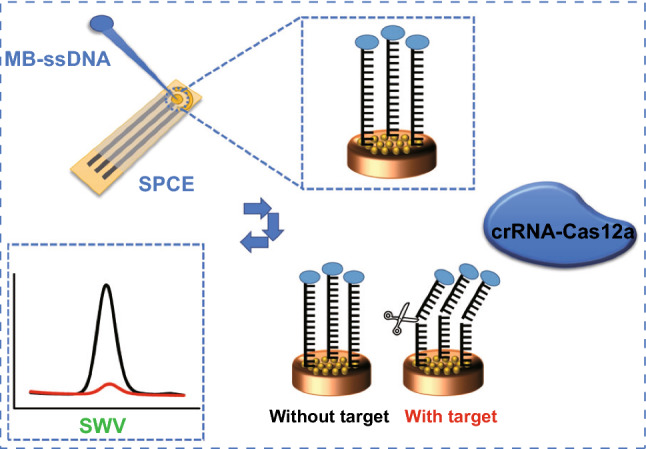

## Introduction

The global outbreak of coronavirus disease 2019 (COVID-19) caused by the severe acute respiratory syndrome coronavirus 2 (SARS-CoV-2) has spread rapidly all over the world [1–3]. With 525,467,084 confirmed cases and 6,285,171 deaths worldwide, by 27 May 2022 (WHO Coronavirus (COVID-19) Dashboard, 2022), the world is suffering a considerable health, social, and economic burden. Consequently, quick and precise identification and monitoring of coronavirus infection are crucial for preventing disease transmission and ultimately saving lives [4–7]. Generally, there are two diagnostic methods for SARS-CoV-2, serological and viral nucleic acid tests [8–11]. Serological testing detects the presence of antibodies or the antigenic viral proteins produced by an (infected) individual because of exposure to the virus. The antibody test should not be used to diagnose people with active infections and may give false-negative results because the body’s immune system may not be active in the early stage of infection [8, 9, 12]. Rapid antigen tests for SARS-CoV-2 are cost-effective and give immediate results, while the tests are generally not as sensitive as nucleic acid-based tests. Consequently, to accurately diagnose active SARS-CoV-2 infection, viral nucleic acid testing should be used. Currently, the gold standard method for diagnosing SARS-CoV-2 is a diagnostic test based on the detection of viral RNA by quantitative reverse transcription polymerase chain reaction (qRT-PCR) [13–15]. However, there are some disadvantages of qRT-PCR, such as being time-consuming and requiring a specialized laboratory setting with expensive instruments and trained personnel [14–16].

Since the first application of the CRISPR-Cas9 system for gene editing in the mammalian genome, CRISPR-Cas systems have exploded in the field of biotechnology and have become an essential tool for transcriptional regulation and genome editing, among other applications [17–20]. Recently, CRISPR-Cas effectors (*e.g.,* Cas12, Cas13) have been deployed in nucleic acid detection, which show great potential in terms of novel biosensors fabrication for nucleic acid detection due to their unique properties of providing signal output when target nucleic acids and single-stranded non-targeted nucleic acids (employed as reporters) are cleaved [18, 20]. There are many different types of Cas nuclease that have been applied in gene detecting methodologies. For example, by employing Cas12 or Cas13 and pre-amplifying DNA or RNA sequences using RPA, Zhang et al. designed SHERLOCK [21], a gene detecting method with high specificity and sensitivity. As SHERLOCK initiated the rapid development of CRISPR-based diagnostics (CRISPR-Dx), the following methods like HOLMES [22, 23], HOLMESv2 [24], CONAN [25], etc., target different types of nucleic acid samples with different methods of amplification. Based on such techniques, Different CRISPR-Dx platforms have been applied in clinical to detect bacteria [26], diagnosis of hereditary diseases [27], and screening of viruses [28]. Now the evolving CRISPR-Dx platforms for SARS-CoV-2 has been reported broadly, it has been determined that the majority of them consist of viral purification, amplification, and detection processes [29–32]. Although these diagnostic methods are making SARS-CoV-2 testing more available to meet the needs of rapid testing, they still rely on the amplification of viral RNA templates, leading to extended experimental time [33], high-cost and sophisticated devices. Recently, there are more and more CRISPR-Dx platforms that avoid the use of polymerase-mediated amplification by improving the sensitivity of detection systems, including droplet microfluidics [34] and modular catalytic hairpin assembly circuits [25, 35]. In addition, CRISPR can also integrate different platforms for enhanced signals. A CRISPR-based surface-enhanced Raman scattering (SERS) assay can detect ~ 10 fM genomic DNA with the target sequence [36]. A graphene field-effect transistor coupling with CRISPR-Cas9 system, as a specific capturer for specific exons of genomic DNA related to inherited disease [27]. Our group also designed a CRISPR-empowered surface plasmon resonance (SPR) platform, fulfilling accurate detection of ~ 5 fM unamplified DNA samples [37].

Electrochemical biosensors have attracted considerable attention as powerful analytical tools in medical diagnostics, because they offer various advantages over other diagnostic procedures in diagnostics, such as high sensitivity, simplicity, rapid response, and cost-effectiveness [38, 39]. Among the currently available nucleic acid-based biosensors, electrochemical biosensors have shown their powers for POCT applications as well [40–42]. In addition, gold nanoparticle (AuNP) has high biocompatibility, low toxicity, and well-established biochemistry application. It is one of the most popular and robust selection for biosensor fabrication [43, 44]. Therefore, in this work, we first present the methodology of electrochemical CRISPR sensing (MOECS), to address the demand for rapid and accurate detection of the variants of SARS-CoV-2. For enhanced sensing performance, the working electrode (AuE) is modified with AuNPs to increase the conductivity and specific surface area by electrodeposition because the deposition of high-quality AuNPs by *in situ* electro-deposition method is simple in operation and can be completed in a few hundred seconds. After that, the AuNPs decorated AuE (AuE-AuNPs) is modified by methylene blue-single stranded DNA (MB-ssDNA) which acts as reporter gene. For stable electrochemical signal and long-time storage, Cas12a protein was chosen to fabricate the biosensor. When the biosensor is treated by Cas12a-crRNA-target DNA triplex, the trans-cleavage activity of Cas12a is activated and the MB-ssDNA would be un-specifically cleaved off from the electrode surface. Therefore, the electron transfer between the AuE and the redox mediator (MB) on the ssDNA is altered before and after the cleavage, while the variation can be electrochemically transduced and detected. The feasibility of developed electrochemical biosensor based on the CRISPR-Cas system (E-CRISPR) for SARS-COV-2 Delta variant was examined by both biological and electrochemical strategies. Accordingly, the limit of detection (LOD), specificity and stability of the biosensor are characterized. Finally, the performance and potential application in POCT of the E-CRISPR are explored on the micro-electrochemical platform.

## Experimental Section

### Materials and Instruments

NaOH, H_2_SO_4_, KCl, NaCl, MgCl_2_, HAuCl_4_, Tris–HCl, K_3_[Fe(CN)_6_], K_4_[Fe(CN)_6_], Tris-(2-carboxyethyl) phosphine hydrochloride (TCEP), 6-mercaptohexanol (MCH), Ethylenediaminetetra acetic acid (EDTA) (all analytical grade) were purchased from Macklin Biochemical Co., Ltd (Shanghai, China). Primer sets were synthesized by the Shanghai Generay Biotech Co., Ltd (China). Other oligonucleotides, including the crRNA, MB-ssDNA reporter, and FAM-ssDNA reporter, were synthesized by Sangon Biotech (Shanghai) Co., Ltd. (Shanghai, China). Their sequences are listed in Table S1.

Electrochemical measurements were performed on CHI 760E electrochemical workstation (Shanghai Chenhua Instrument Co. Ltd., China). Gold electrode (AuE, 3 mm in diameter), Ag/AgCl electrode, and platinum (Pt) wire were employed as working, reference, and counter electrodes in three-electrode system, respectively, in which the working electrode can be reused thousands of times. Screen-printed electrode (SPEC) and micro-electrochemical workstation for POCT assays were obtained from Shenzhen Refresh Intelligent Technology Co, Ltd., China. The morphology of the electro-deposited AuNPs was in situ characterized by scanning electron microscopy (TESCAN MIRA LMS).

A Cary Eclipse fluorescence spectrophotometer (Agilent Technologies, Palo Alto, CA) was used to read the fluorescence spectra. Concentrations of DNA suspensions were quantified by using NanoDrop 1000 spectrophotometer (Thermo Scientific, USA). The analysis of agarose gel electrophoresis was performed by an electrophoresis analyzer (Bio-Rad, USA) and imaged with a ChemiDoc XRS system (Bio-Rad, USA).

### Artificial Synthesis of SARS-CoV-2 Nucleic Acid Fragments

For detecting the Delta variant, part of the spike (S) gene (Table S2) was selected as the target sequence because D950N (24410 G˃A) mutation is a featured mutation for Delta and has not been found in any other variants yet. Based on the adjacent sequence of the mutation sites, specific crRNA is synthesized (Table S1).

Cas12a crRNA consists of two main parts: the universal scaffold region (UAAUUUCUACUAAGUGUAGAU) for Cas12a protein to recognize and bind. The other part is a customized region, which is identical to the sequence of the target site (17–20 nt), extended from the 3′ end of the scaffold and ensures the specificity. As the dsDNA templates, plasmids containing wild-type or mutated S gene sequences, MERS, Influenza virus, and HRSV (Table S2) were also synthesized. All the crRNA and plasmids are provided by Sangon Biotech (Shanghai) Co., Ltd. Besides, nucleic acid templates from SARS-CoV-2 virus (L02087A, Genscript, Nanjing, China) were extracted by using a virus RNA extraction kit (4992285, TIANGEN, Beijing, China) according to the manufacturer’s instruction, and reverse-transcribed into the cDNA template.

### Fabrication of the ssDNA-Modified Electrode

Before modification, the bare AuE was carefully polished by using alumina powder (0.3 and 0.05 μm in diameter, respectively) to obtain a mirror-like surface followed by sonicating with ethanol and deionized water, respectively. After that, the electrochemical cleaning procedure was performed to further remove the oxides and impurities on the surface of the AuE. In detail, a series of cyclic voltammetry (CV) cycles (0.1 V s^−1^, from − 1 to 1 V vs. Ag/AgCl) in 0.5 M NaOH, 0.5 M H_2_SO_4_, 0.1 M H_2_SO_4_ with 0.01 M KCl and 0.05 M H_2_SO_4_ with 0.01 M KCl were applied, respectively, until repeated CV curves were obtained. After washing with deionized water, the AuE was dried with nitrogen for further use. The AuNPs modified AuE was fabricated by an electrochemical deposition method. The electrodeposition on the pretreated AuE was conducted at 0.5 V vs Ag/AgCl in a stable 5 mM HAuCl_4_ solution (pH 5.0) for 300 s by chronoamperometry.

For sample analysis, 100 μM thiolated MB-ssDNA reporters were pretreated with 10 mM TCEP in the dark at 37 °C for 2 h to reduce the thiol–thiol bonds. After that, the MB-ssDNA reporter was diluted into 1 μM by adding Tris-buffer solution (10 mM Tris–HCl, 2 mM EDTA, 10 mM MgCl_2_, 0.1 M NaCl, pH 7.4). And then, 10 μL of the reduced and diluted MB-ssDNA reporter was directly incorporated onto the pretreated AuE-AuNPs surface and incubated in the dark at 37 °C for 4 h in humidity. The MB-ssDNA modified AuE was then rinsed by 10 mM Tris–HCl buffer (pH 7.4). The cleaned MB-ssDNA modified AuE-AuNPs was then immersed into 10 mM Tris–HCl solution (pH 7.4) containing 1 mM MCH for 1 h to passivate the surface and obtain well-aligned DNA monolayer. Finally, after thoroughly washing with 10 mM Tris–HCl buffer (pH 7.4), the electrode was dried with nitrogen and ready for the following processes. Note that: For short-term storage, the modified AuE can be stored at 4 °C in the dark under nitrogen protection.

### Electrochemical Detection of Artificial Samples

For electrochemical analysis of trans-cleaved reporters, the Cas12a-mediated cleavage assay was carried out by adding a Cas12a-crRNA duplex (100 nM Cas12a, 100 nM crRNA, 1× NEBuffer 2.1) into target DNA to form Cas12a-crRNA-target DNA triplex. Then, the triplex was dropped onto the MB-ssDNA modified AuE and incubated at 37 °C for 45 min in humidity. After Cas12a-mediated cleavage treatment, the AuE was thoroughly rinsed by 10 mM Tris–HCl buffer (pH 7.4) and then dried with nitrogen before the following electrochemical processes.

The electrochemical square wave voltammetry (SWV) was performed in 8 mL of 10 mM Tris buffer (pH 7.4) containing 0.1 M NaCl with a potential increment of 4 mV, a frequency of 25 Hz, an modulation amplitude of 25 mV, and a potential range from − 0.45 to 0 V vs. Ag/AgCl. The Δ*I* (%) used for quantitative analysis can be calculated as $\frac{{I}_{0}-I}{{I}_{0}}$, in which *I*_*0*_ is the current before cleavage (without target DNA), *I* represents the current after cleavage (with target DNA). EIS operating conditions were as follows: 0.1 M KCl solution contenting 5 mM [Fe(CN)_6_]^3−/4−^, biased potential of 0.23 V (vs. Ag/AgCl) in the frequency range of 0.01–10^5^ Hz, and 5 mV amplitude.

### Analysis of Mismatch, Interferential Virus

Specificity of crRNAs to different sequences from the samples was validated by performing a typical CRISPR-Cas12a diagnosis assay: each 10 μL reaction mix contains 100 nM Cas12a (NEB), 100 nM crRNA, 1× NEB 2.1 buffer (NEB), 500 nM single-stranded DNA (ssDNA) fluorescent quenched reporter (5′ 6- FAM/TTATTATT/BHQ-1 3’, Sangon), and 10 nM target dsDNA sequences, including wild-type or Delta S gene sequences, MERS, Influenza virus, or HRSV. were diluted in Rnase/Dnase-free water into 10 μL. Reactions were incubated at 37 °C for 30 min. Real-time fluorescence was measured using a BioTek NEO HTS plate reader (BioTek Instruments) with readings every 2 min (excitation: 485 nm; emission: 528 nm). The cis-cleavage of sequences was further verified by electrophoresis on 2.5% agarose gel under 120 V for 30 min.

## Results and Discussion

**Fig. 1:**
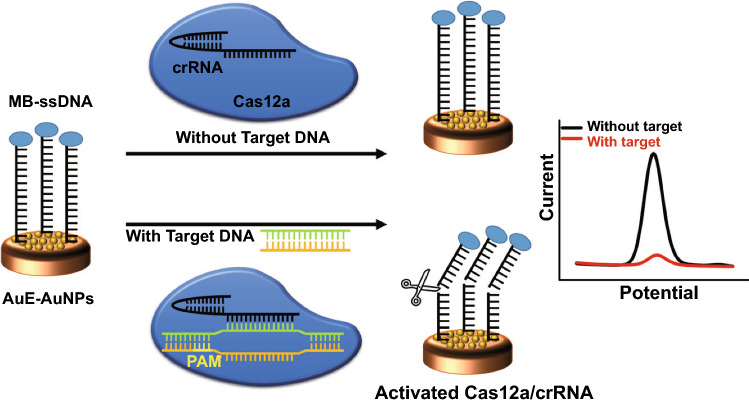
Schematic representation of the AuNPs-assisted E-CRISPR biosensor

The principle of the electrochemical biosensor for the detection of SARS-COV-2 Delta variant based on CRISPR-Cas12a-mediated nonspecific cleaving of MB-ssDNA reporter is illustrated in Fig. 1. First, the bare AuE was decorated by AuNPs by electrodeposition before the immobilization of the MB-ssDNA reporter. Next, the guide Cas12a/crRNA duplex was designed to specifically recognize the target DNA of SARS-COV-2 based on the protospacer adjacent motif (PAM) sequence of the target and the complementarity between target DNA and crRNA [45]. In the absence of the target DNA, the cleavage activity of the Cas12a-crRNA was not activated and the MB-ssDNA reporters were retained on the modified electrode surface, resulting in a distinct electrochemical signal of MB. In the presence of the target DNA, the Cas protein recognizes the PAM sequence and the Cas protein as DNA helicase unwinds the target DNA. After activating the trans-cleavage activity of Cas12a, the MB-ssDNA reporters were nonspecifically cleaved off from the electrode surface, resulting in a low electrochemical signal of MB [46]. Therefore, the MB-ssDNA immobilized AuE-AuNPs realized the electrochemical transduction of the CRISPR detection signal based on the transduction of the electron transfer between AuE-AuNPs and MB on ssDNA. Furthermore, the fabricated AuNPs-assisted E-CRISPR could convert target recognition events into massive cleavage of the ssDNA reporter on the electrode for highly sensitive electrochemical nucleic acid biosensing.

**Fig. 2:**
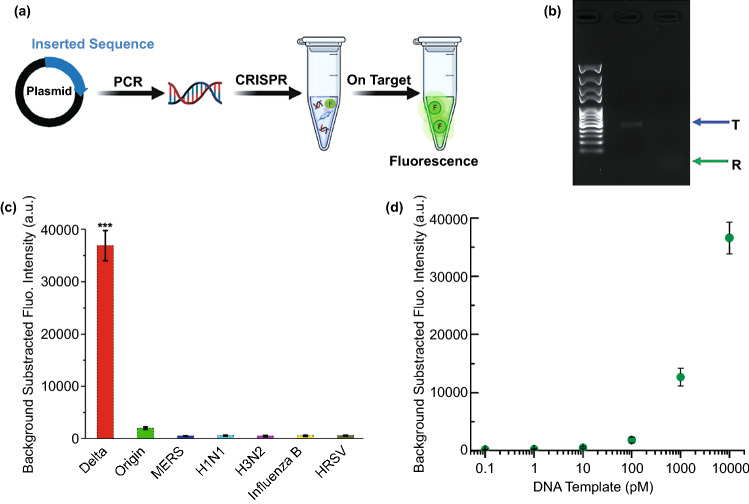
Feasibility characterization of the CRISPR system. **a** Streamline the CRISPR reaction. **b** Validation of trans- and cis-cleavage through agarose gel electrophoresis. **c** Specificity of the designed crRNA. **d** Fluorescence-concentration results, the grey line indicates the limit of detection. All the experiments were performed in triplicate, and the significance was presented as ***P < 0.001

To examine the feasibility of a CRISPR-Cas12a-based electrochemical biosensor for nucleic acid detection, experiments were performed before the on-device detection. As shown in Fig. 2a, different target templates were designed and inserted into pUC57 plasmids. Through the amplification using PCR, DNA templates were obtained and the concentrations were determined. CRISPR detection was performed using a Cas12a-crRNA complex, and a FAM-BHQ ssDNA reporter. Only if the dsDNA template contains an identical part of crRNA could the Cas12a protein be activated, thus cleaving the template (cis-cleavage) and the ssDNA reporter (trans-cleavage), then the freed FAM generates fluorescence to be detected.

The cis- and trans-cleavage was confirmed using agarose gel electrophoresis. As shown in Fig. 2b, the band of intact dsDNA template was observed (indicates as “T”). After the CRISPR reaction with the right crRNA, the band of the template disappeared (cis-cleavage), and fluorescence of the reporter was observed (“R” band, trans-cleavage). A photograph of the gel under ultraviolet can be found in Fig. S1. In this study, the target DNA of SARS-COV-2 variants, influenza viruses, and HRSV were chosen to prove the specificity of the crRNA used to detect the Delta variant of SARS-CoV-2. As shown in Fig. 2c, only the DNA template of the Delta variant reacted with the CRISPR system and strong fluorescence was observed. Other DNA templates of some respiratory viruses, including the wild-type (Origin) SARS-CoV-2, only significantly weaker (*P* < 0.001) fluorescence was observed. Furthermore, FAM-ssDNA-BHQ probe (Table S1) was used as reporter of Cas12a for fluorescence detection of the target Delta DNA with different concentrations (from 100 fM to 10 nM), and the LOD was calculated to be about 100 pM (Figs. 2d and S2).

Next, the E-CRISPR for SARS-COV-2 Delta variant was characterized by electrochemical methods to investigate the cleavage feasibility and sensitivity. At the beginning of electrode modification, AuNPs were electro-deposited on the surface of the bare AuE to form AuE-AuNPs with higher specific surface area and electrical conductivity [47]. The average diameter of the electrochemically deposited AuNPs is about 50 nm, and AuE was covered by the homogeneous distributed AuNPs (Fig. S3). As expected, the electrochemical surface area of AuE (2.17 mm^2^) and AuE-AuNPs (12.3 mm^2^) were calculated by integrating the gold oxide reduction peak at around 0.1 V vs Ag/AgCl in Fig. S4A, which confirmed the larger active surface area of AuE-AuNPs (about 5.7 times to AuE) [48]. In addition, EIS of bare AuE and AuE-AuNPs were carried out because EIS was highly effective for evaluating the interfacial electron transfer efficiency at different stages in biosensor fabrication [49]. The electron transfer resistance (*R*_et_) is the main indicator and corresponds to the semicircle diameter in the Nyquist diagrams [18]. The EIS characterization showed that the *R*_et_ of bare AuE was about 180 Ω in high frequency, while the Nyquist diagram of AuE-AuNPs was an almost straight line without semicircle (*R*_et_ close to 0 Ω), indicating the higher electrochemical conductivity of AuE-AuNPs than bare AuE (Fig. S4B) [50]. After the immobilization of MB-ssDNA on the surface of bare AuE and AuE-AuNPs, the square wave voltammetry (SWV) was performed to characterize the quality of the biosensor as well. Clearly, the redox peak of MB at around − 0.27 V vs. Ag/AgCl on AuE-AuNPs was much higher than that on AuE (Fig. S5), which demonstrated that a large amount of MB-ssDNA molecules was immobilized on the surface of AuE-AuNPs because of the enlarged active surface area by AuNPs [51]. Therefore, the AuE-AuNPs would increase the immobilization efficiency of the reporter ssDNA and subsequently the efficient activity of trans-cleavage.

**Fig. 3:**
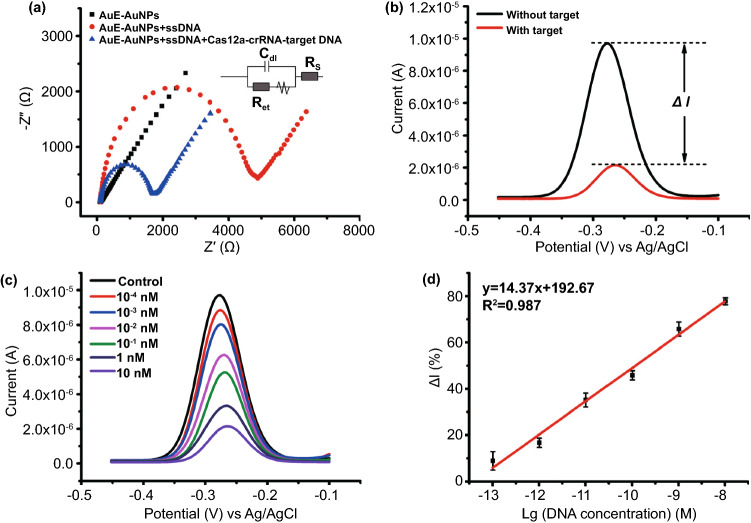
Feasibility and sensitivity analysis of the E-CRISPR biosensor by electrochemical method. **a** Electrochemical impedance spectroscopy (EIS) of AuE-AuNPs (black curve), ssDNA modified AuE-AuNPs (red curve) and ssDNA modified AuE-AuNPs after Cas12a cleavage (blue curve). **b** Square wave voltammetry (SWV) curves of ssDNA modified AuE-AuNPs without (black curve) and with (red curve) target. **c** SWV curves for a range of target DNA from 100 fM to 10 nM (red curve to purple curve), control refers to the replacement of target DNA with H_2_O. **d** Linear relationship between the change of current (*ΔI%*) and the logarithm of the target DNA concentration. Error bars represent standard derivations obtained in three parallel experiments

After that, EIS of the biosensor at different stages were carried out to verify the successful fabrication of E-CRISPR for SARS-COV-2. As displayed in Fig. 3a, the AuE-AuNPs possessed an excellent electrochemical conductivity (black curve). After the fix of MB-ssDNA on the AuE-AuNPs, the value of *R*_et_ increased to 4795 Ω dramatically (red curve), which could be attribute to the electrostatic repulsive force with [Fe(CN)_6_]^3−/4−^ and increased electron transfer distance caused by the self-assembled negatively charged MB-ssDNA monolayer [49, 51]. After treated with the Cas12a-crRNA-target DNA triplex, most of the MB-ssDNA has been cleaved and separated from the AuE-AuNPs surface because of the activated cleavage activity of Cas12a. As expected, the *R*_et_ showed considerable decrease (about 1,612 Ω) in the blue curve. The results demonstrated the successful modification and the target-induced cleavage of MB-ssDNA on AuE-AuNPs surface [52]. Square wave voltammetry (SWV) was applied to evaluate the feasibility of the biosensor. As revealed in Fig. 3b, a high redox peak of MB at about − 0.27 V vs. Ag/AgCl was observed when the biosensor was treated with Cas12a-crRNA duplex and without target DNA (black curve). While a dramatically decreased redox peaks of MB was presented when the biosensor was treated with Cas12a-crRNA together with target DNA (red curve), which further confirmed the successful cleavage of MB-ssDNA and release of MB on the biosensor surface [18, 52]. *ΔI* represented the different value between the current before and after cleavage. All in all, these results demonstrated that the fabricated AuNPs-assisted E-CRISPR could be applied for nucleic acid detection.

Afterwards, the sensitivity of the constructed SARS-COV-2 Delta variant biosensor was estimated. Figure 3c shows the SWV of the redox peaks of MB with the target DNA concentration range from 100 fM to 10 nM, in which a gradual current decrease was observed as the target DNA concentration increased. Besides, the *ΔI%* versus the logarithm of target DNA concentration showed a good correlation in the investigated concentration range (Fig. 3d). The regression equation was Δ*I*% = 14.37 lgC + 192.67, the *R*^2^ and the LOD were calculated to be 0.987 and 50 fM (3σ rule) [49, 51], which indicated a commendable linearity and ultra-high sensitivity, respectively. Generally, the cleavage of ssDNA and detection process can be rapid finished within 1 h. Besides, the LOD of fluorescence detection of the target Delta DNA was calculated to be 100 pM, which is much higher than that of the electrochemical biosensor (Figs. 2d and S2). Clearly, E-CRISPR should be a robust strategy for the detection of SARS-COV-2 without amplification, which deserves more attention and discussion.

**Fig. 4:**
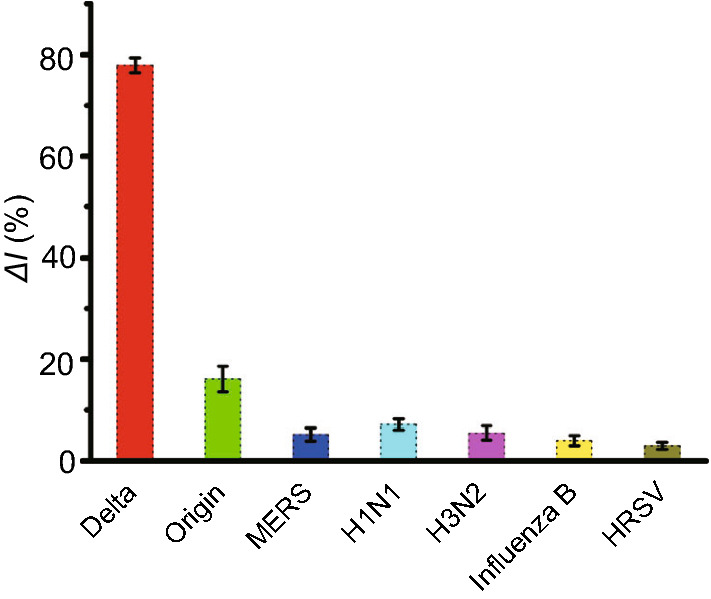
Specificity analysis of the AuNPs assisted E-CRISPR for SARS-COV-2 Delta variant. The change in signal was calculated based on the SWV current with the addition of the target DNA (10 nM) and non-target virus (10 nM), respectively. Error bars represent standard derivation obtained in three parallel experiments

Furthermore, the specificity and selectivity of the E-CRISPR for SARS-COV-2 Delta variant wereinvestigated by testing other nucleus acid extracted from the original SARS-CoV-2, MERS, and some other respiratory tract infection related virus, which should not appears cross-reaction signal. In the reaction system that detecting Delta variant, gene sequence of origin SARS-CoV-2 lacks of a PAM sequence. PAM is not a complementary part of the protospacer, but it is a crucial sequence for the activation of Cas12a protein [53]. As expected in Fig. 4, a low Δ*I*% was observed when the target DNA was replaced by the nucleus acid of origin SARS-CoV-2 (16.1%), which confirmed the specificity of crRNA targeting the Delta variant. Besides, MERS, H1N1, H3N2, Influenza B and HRSV all showed unconspicuous signal changes (Δ*I*% < 10%), while only the target DNA from Delta variant exhibited significant electrochemical responses (Δ*I*% = 77.9%). These results demonstrated that the established detection platform was capable of testing the SARS-COV-2 Delta variant with high specificity. Meanwhile, due to the programmability of crRNA, Cas-based biosensors allow the development of a general biosensing platform for any other emerging SARS-CoV-2 variants by easily changing the crRNA guide region sequence in theoretical [54, 55]. Moreover, the long-time stability of in vitro diagnostic devices is critical for practical applications. Hence, the stability and reproducibility of the AuNPs-enforced E-CRISPR platform have been characterized. The MB-ssDNA modified electrodes were stored in a dry box under nitrogen at 4 °C. The SWV signal remained stable (the signal decreased < 10%) within a week (Fig. S6), which is a sufficient turnaround time for the detection of SARS-COV-2.

**Fig. 5:**
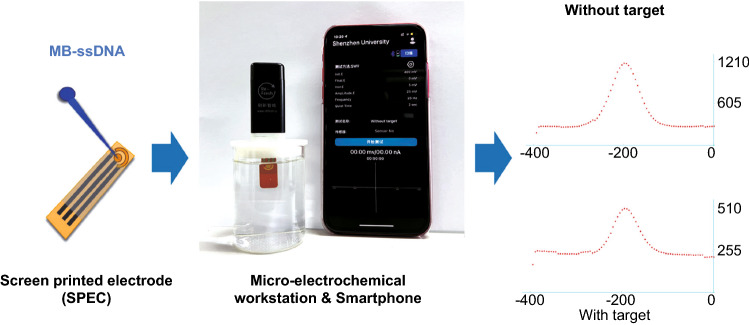
Schematic illustration, primary assay photograph and experimental results of the E-CRISPR application in POCT for SARS-CoV-2 Delta variant detection

The POCT eliminates dependence on large instruments and making analysis and detection more flexible and convenient [56]. At the same time, SARS-CoV-2 demands POCT in particular due to its rapidly transmissible nature [13]. Therefore, the established E-CRISPR for SARS-CoV-2 variant was transferred into the wireless micro-electrochemical platform to explore the potential application in POCT. The schematic principle, photograph of primary assay and experimental results are shown in Fig. 5, in which the micro-electrochemical workstation was connected and directly controlled by the smartphone and the experimental data could be transported by Bluetooth in time. The work electrode of customed SPEC is gold with 3 mm in diameter, while the counter electrode is gold and the reference electrode is Ag/AgCl (Fig. S7). Therefore, the assays in this work based on the AuE were transferred and carried out on the SPEC. The screen-printed technology is mature for the preparation of SPEC till now; thus, the cost of SPEC for POCT is ultra-low. Considering the system differences between the traditional electrochemical workstation and the micro-electrochemical workstation, the results should be distinct [57]. And the concentration of the target DNA in POCT assay was chosen as 10 nM just to prove the feasibility of the detection of SARS-COV-2 variant by micro-electrochemical workstation for POCT.

As shown in Fig. 5, the redox peak currents of MB from the SWV curve were 933.05 nA (without target DNA) and 280.11 nA (with target DNA), respectively, Δ*I*% was calculated to be 69.97%, which was only a 10% difference compared with the corresponding Δ*I*% obtained from the traditional electrochemical workstation. Clearly, the E-CRISPR combined with micro-electrochemical platform can be applied to detect SARS-CoV-2 Delta variant as a POCT for quick and simple diagnosis without troublesome sample handling. Thanks to the popularity of smartphones, the development of wireless connection technology, and portable micro-electrochemical platform, the great potential of CRISPR-Cas-based biosensor for future industrialization prospects is promising.

## Conclusions

In summary, MOECS combined the advantages of electrochemical sensor (rapid and high sensitivity) and CRISPR-Cas system (high specificity), which is urgent for the detection of SARS-CoV-2 Delta variant. In the novel MOECS-based biosensor, uniform AuNPs deposited on the AuE enhanced the sensing performance by improving conductivity and enlarging the electrochemical active surface area. Meanwhile, benefiting from the high-efficiency trans-cleavage activity of Cas12a-Cas system, the optimized electrochemical performance showed a wide linear range from 100 fM to 10 nM with high linearity (*R*^2^ = 0.987) and an ultralow LOD of 50 fM. The detection can be finished in 1 h and the E-CRISPR displayed exceptional specificity and long-time stability. As a comparable supplement for the RT-qPCR method, this CRISPR-Cas12a-based electrochemical biosensor will possess profound significance for the early diagnosis of SARS-CoV-2 in a pandemic situation and simultaneously hold universality and scalability in other SARS-CoV-2 variants detection. One more highlight is that the POCT application based on the wireless micro-electrochemical platform successfully inherits the remarkable advantages (rapidness, cost-efficient, and simple operation) of the E–CRISPR. has been successfully expanded to the POCT application based on the wireless micro-electrochemical platform. In summary, the proposed of MOECS enables the fabrication of high quality biosensor for the detection of SARS-CoV-2 variants, which should be further developed and applied to other variants and clinical samples in the upcoming future.

## Supplementary Information

Below is the link to the electronic supplementary material.

Supplementary file 1 (PDF 521 KB)
