# Supplementary material for: CRISPR-Cas12a-Empowered Electrochemical Biosensor for Rapid and Ultrasensitive Detection of SARS-CoV-2 Delta Variant
Source: Nanomicro Lett. 2022 Aug 4;14:159. doi: 10.1007/s40820-022-00888-4 (PMC9352833; doi:10.1007/s40820-022-00888-4)
Supplement: Supplementary file 1 — (PDF 521 KB) [file 40820_2022_888_MOESM1_ESM.pdf]

Supporting Information for

CRISPR-Cas12a-Empowered Electrochemical Biosensor for Rapid and Ultrasensitive Detection of SARS-CoV-2 Delta Variant

Chenshuo Wu[1], Zhi Chen[1,*], Chaozhou Li[1], Yabin Hao[1, 5], Yuxuan Tang[1, 6], Yuxuan Yuan[1], Luxiao Chai[1], Taojian Fan[1], Jiangtian Yu[2], Xiaopeng Ma[3], Omar A. Al-Hartomy[7], S. Wageh[7], Abdullah G. Al-Sehemi[8, 9], Zhiguang Luo[10], Yaqing He[11], Jingfeng Li[1, 2, *], Zhongjian Xie[2, 4, *], Han Zhang[1, *]

[1] International Collaborative Laboratory of 2D Materials for Optoelectronics Science and Technology of Ministry of Education, Institute of Microscale Optoelectronics, College of Physics and Optoelectronic Engineering, Shenzhen University, Shenzhen 518060, P. R. China

[2] Shenzhen International Institute for Biomedical Research, Shenzhen 518116, P. R. China

[3] Department of Respiratory, Shenzhen Children's Hospital, Shenzhen 518038, P. R. China

[4] Institute of Pediatrics, Shenzhen Children's Hospital, Shenzhen 518038, P. R. China

[5] Shenzhen Han's Tech Limited Company, Shenzhen 518000, P. R. China

[6] Shenzhen Metasensing Tech Limited Company, Shenzhen 518000, P. R. China

[7] Department of Physics, Faculty of Science, King Abdulaziz University, Jeddah 21589, Saudi Arabia

[8] Research Center for Advanced Materials Science (RCAMS), King Khalid University, P.O. Box 9004, Abha 61413, Saudi Arabia

[9] Department of Chemistry, College of Science, King Khalid University, P.O. Box 9004, Abha 61413, Saudi Arabia

[10] Zhongmin (Shenzhen) intelligent ecology Co., Ltd. Shenzhen 518055, P. R. China

[11] Shenzhen Center for Disease Control and Prevention, Shenzhen 518055, P. R. China

*Corresponding authors. E-mail: alexchen017010913@yahoo.com (Zhi Chen); ljfeng0623@163.com (Jingfeng Li); zjxie@siitm.org.cn (Zhongjian Xie); hzhang@szu.edu.cn (Han Zhang)

Supplementary Tables and Figures

Table S1 crRNA and reporters used in this study. crRNA is designed according to "CRISPR-SHERLOCK" method published by Feng Zhang *et al*. [S1]

Name	Sequence (5'-3')
crRNA-Delta	UAAUUUCUACUAAGUGUAGAUAAGTTTTCCAAGTGCACTTG
MB-ssDNA reporter	SH-CTCAACttattattACGAAC-MB
FAM-ssDNA reporter	FAM- CTCAACttattattACGAAC-BHQ

Table S2 Sequences from SARS-CoV-2 (Origin/Delta), Middle East respiratory syndrome-related coronavirus (MERS), Influenza A virus (H1N1/H3N2), Influenza B virus, Human Respiratory Syncytial Virus (HRSV). In the sequence of SARS-CoV-2 Origin, the mutation site labelled in red indicates the specific mutation in Delta variant (D950N: 24410 G > A)

Name	Inserted Sequences (5'-3')
SARS-CoV-2 Origin<br>(NC_045512.2, S gene, 24192-24643)	TGTTAGCGGGTACAATCACTTCTGGTTGGACCTTTGGTGCAGGTGCT GCATTACAAATACCATTTGCTATGCAAATGGCTTATAGGTTTAATGG TATTGGAGTTACACAGAATGTTCTCTATGAGAACCAAAAATTGATTG CCAACCAATTTAATAGTGCTATTGGCAAAATTCAAGACTCACTTTCT TCCACAGCAAGTGCACTTGGAAAACTTCAAG(Delta:G>A)ATGTGGTC AACCAAAATGCACAAGCTTTAAACACGCTTGTTAAACAACTTAGCTC CAATTTTGGTGCAATTTCAAGTGTTTTAAATGATATCCTTTCACGTCT TGACAAAGTTGAGGCTGAAGTGCAAATTGATAGGTTGATCACAGGC AGACTTCAAAGTTTGCAGACATATGTGACTCAACAATTAATTAGAGC TGCAGAAATCAGAGCTTCTGCTAATCTTGCTGCTACT
MERS (NC_019843.3, 24275-24938)	TGTTAATATGGAAGCCGCGTATACTTCATCTTTGCTTGGCAGCATAG CAGGTGTTGGCTGGACTGCTGGCTTATCCTCCTTTGCTGCTATTCCAT TTGCACAGAGTATCTTTTATAGGTTAAACGGTGTTGGCATTACTCAA CAGGTTCTTTCAGAGAACCAAAAGCTTATTGCCAATAAGTTTAATCA GGCTCTGGGAGCTATGCAAACAGGCTTCACTACAACTAATGAAGCT TTTCAGAAGGTTCAGGATGCTGTGAACAACAATGCACAGGCTCTATC CAAATTAGCTAGCGAGCTATCTAATACTTTTGGTGCTATTTCCGCCT CTATTGGAGACATCATACAACGTCTTGATGTTCTCGAACAGGACGCC CAAATAGACAGACTTATTAATGGCCGTTTGACAACACTAAATGCTTT TGTTGCACAGCAGCTTGTTCGTTCCGAATCAGCTGCTCTTTCCGCTCA ATTGGCTAAAGATAAAGTCAATGAGTGTGTCAAGGCACAATCCAAG CGTTCTGGATTTTGCGGTCAAGGCACACATATAGTGTCCTTTGTTGT AAATGCCCCTAATGGCCTTTACTTCATGCATGTTGGTTATTACCCTA GCAACCACATTGAGGTTGTTTCTGCTTATGGTCTTTGCGATGCAGCT AACCCT
Influenza A virus, H1N1<br>(NC_026431.1)	ATGAGTCTTCTAACCGAGGTCGAAACGTACGTTCTTTCTATCATCCC GTCAGGCCCCCTCAAAGCCGAGATCGCGCAGAGACTGGAAAGTGTC TTTGCAGGAAAGAACACAGATCTTGAGGCTCTCATGGAATGGCTAA AGACAAGACCAATCTTGTCACCTCTGACTAAGGGAATTTTAGGATTT GTGTTCACGCTCACCGTGCCCAGTGAGCGAGGACTGCAGCGTAGAC GCTTTGTCCAAAATGCCCTAAATGGGAATGGGGACCCGAACAACAT GGATAGAGCAGTTAAACTATACAAGAAGCTCAAAAGAGAAATAAC GTTCCATGGGGCCAAGGAGGTGTCACTAAGCTATTCAACTGGTGCA CTTGCCAGTTGCATGGGCCTCATATACAACAGGATGGGAACAGTGA CCACAGAAGCTGCTTTTGGTCTAGTGTGTGCCACTTGTGAACAGATT GCTGATTCACAGCATCGGTCTCACAGACAGATGGCTACTACCACCA ATCCACTAATCAGGCATGAAAACAGAATGGTGCTGGCTAGCACTAC GGCAAAGGCTATGGAACAGATGGCTGGATCGAGTGAACAGGCAGC GGAGGCCATGGAGGTTGCTAATCAGACTAGGCAGATGGTACATGCA ATGAGAACTATTGGGACTCATCCTAGCTCCAGTGCTGGTCTGAAAGA TGACCTTCTTGAAAATTTGCAGGCCTACCAGAAGCGAATGGGAGTG CAGATGCAGCGATTCAAGTGATCCTCTCGTCATTGCAGCAAATATCA TTGGGATCTTGCACCTGATATTGTGGATTACTGATCGTCTTTTTTTCA AATGTATTTATCGTCGCTTTAAATACGGTTTGAAAAGAGGGCCTTCT ACGGAAGGAGTGCCTGAGTCCATGAGGGAAGAATATCAACAGGAA

	CAGCAGAGTGCTGTGGATGTTGACGATGGTCATTTTGTCAACATAGA<br>GCTAGAGTAA
Influenza A virus, H3N2<br>(U51247.1)	ATGAATCCAAATCAAAAGATAATAACAATTGGCTCTGTTTCTCTCAC<br>TATTGCCACAATATGCTGCCTTATGCAAATTGCCATCCTGGTAACTA<br>CTGTAACATTACATTTCAAGCAATATGAATGCAACTCCCCCCCAAAC<br>AACCAAGTAATGCTGTGTGAACCAACAATAATAGAAAGAAACATAA<br>CAGAGATAGTGTATCTGACCAACACCACCATAGAGAAAGAAGTATG<br>CCCCAAACTAGCAGAATACAGAAATTGGTCAAAGCCGCAATGTAAA<br>ATTACAGGATTTGCACCTTTTTCTAAGGACAATTCAATTCGGCTTTCC<br>GCTGGTGGAGACATTTGGGTGACAAGAGAACCTTATGTGTCATGCG<br>ATCCTGGCAAGTGTTATCAATTTGCCCTTGGACAGGGAACAACACTA<br>AACAACAGGCATTCAAATGACACAGTACATGATAGGACCCCTTATC<br>GAACCCTATTGATGAATGAGTTGGGTGTTCCATTTCATTTGGGAACC<br>AAGCAAGTGTGCATAGCATGGTCCAGCTCAAGTTGTCACGATGGAA<br>AAGCATGGCTGCATGTTTGTGTAACTGGGCATGATGAAAATGCAAC<br>TGCTAGCTTCATTTACGATGGGAGGCTTGTAGATAGTATTGGTTCAT<br>GGTCCAAAAATATCCTCAGGACCCAGGAGTCGGAATGCGTTTGTAT<br>CAATGGAACTTGTACAGTAGTAATGACTGATGGAAGTGCTTCAGGA<br>AGAGCTGATACTAAAATACTATTCATTGAAGAGGGGAAAATCGCTC<br>ATATTAGCCCATTGTCAGGAAGTGCTCAGCATGTCGAGGAGTGCTCC<br>TGTTATCCTCGATATCCTGGTGTCAGATGTGTCTGCAGAGACAACTG<br>GAAAGGCTCCAATAGGCCCATCGTAGATATAAATGTGAAAGATTAT<br>AGCATTGTTTCCAGTTATGTGTGCTCAGGACTTGTTGGAGACACAGC<br>CAGAAAAAACGACAGCTCCAGCAGTAGCTATTGCCGGAATCCTAAC<br>AATGAGAAAGGGAGTCATGGAGTGAAAGGCTGGGCCTTTGATGATG<br>GAAATGATGTGTGGATGGGAAGAACGATCAGCGAGAAGTTACGCTC<br>AGGTGATGAAACCTTCAAAGTCATTGGAGGCTGGTCCAAACCTAAC<br>TCCAAATTGCAGATAAATAGGCAAGTCATAGTTGACAGAGGTAATA<br>GGTCCGGTTATTCTGGTATTTTCTCTGTTGAAGGCAAAAGCTGCATC<br>AATCGGTGCTTTTATGTGGAGTTGATAAGGGGAAGGAAACAGGAAA<br>CTGAAGTCTGGTGGACCTCAAACAGTATTGTTGTGTTTTGTGGCACC<br>TCAGGTACATATGGAACAGGCTCATGGCCTGATGGGGCGGACATCA<br>ATCTCATGCCTATATAAA
Influenza B virus segement 7<br>(NC_002210.1)	AGCAGAAGCACGCACTTTCTTAAAATGTCGCTGTTTGGAGACACAAT<br>TGCCTACCTGCTTTCACTAATAGAAGATGGAGAAGGCAAAGCAGAA<br>CTAGCTGAAAAATTACACTGTTGGTTCGGTGGGAAAGAATTTGACCT<br>AGATTCTGCTTTGGAATGGATAAAAAACAAAAGGTGCCTAACTGAT<br>ATACAAAAAGCACTAATTGGTGCCTCTATATGCTTTTTAAAACCCAA<br>AGACCAAGAAAGAAAAAGGAGATTCATCACAGAGCCCCTGTCAGG<br>AATGGGAACAACAGCAACAAAGAAGAAAGGCCTAATTCTAGCTGA<br>GAGAAAAATGAGAAGATGTGTAAGCTTTCATGAAGCATTTGAAATA<br>GCAGAAGGCCACGAAAGCTCAGCATTACTATATTGTCTTATGGTCAT<br>GTACCTAAACCCTGAAAACTATTCAATGCAAGTAAAACTAGGAACG<br>CTCTGTGCTTTATGCGAGAAACAAGCATCGCACTCGCATAGAGCCCA<br>TAGCAGAGCAGCAAGGTCTTCGGTACCTGGAGTAAGACGAGAAATG<br>CAGATGGTTTCAGCTATGAACACAGCAAAGACAATGAATGGAATGG<br>GAAAGGGAGAAGACGTCCAAAAACTAGCAGAAGAGCTGCAAAACA<br>ACATTGGAGTGTTGAGATCTCTAGGAGCAAGTCAAAAGAATGGAGA<br>AGGAATTGCCAAAGATGTAATGGAAGTGCTAAAACAGAGCTCTATG<br>GGAAATTCAGCTCTTGTGAGGAAATACTTATAATGCTCGAACCACTT<br>CAGATTCTTTCAATTTGTTCTTTCATTTTATCAGCTCTCCATTTCATG<br>GCTTGGACAATAGGGCATTTGAATCAAATAAAAAGAGGGGTAAACT<br>TGAAAATACAAATAAGGAATCCAAATAAGGAGGCAATAAACAGAG<br>AGGTGTCAATTCTGAGACACAATTACCAAAAGGAATCCAAGCCAA

	AGAAACAATGAAGAAAATACTCTCTGACAACATGGAAGTATTGGGT<br>GACCACATAGTAGTTGAAGGGCTTTCAACTGATGAGATAATAAAAA<br>TGGGTGAAACAGTTTTGGAGGTGGAAGAATTGCAATGAGCCCAATT<br>TTCACTGTATTTCTTACTATGCATTTAAGCAAATTGTAATCAATGTCA<br>GTGAATAAAACTGGAAAAAGTGCGTTGTTTCTACT
HRSV, Human orthopneumovirus Subgroup A, (NC_038235.1)	GGGGCAAATACAAAGATGGCTCTTAGCAAAGTCAAGTTGAATGATA<br>CACTCAACAAAGATCAACTTCTGTCATCCAGCAAATACACCATCCAA<br>CGGAGCACAGGAGATAGTATTGATACTCCTAATTATGATGTGCAGA<br>AACACATCAATAAGTTATGTGGCATGTTATTAATCACAGAAGATGCT<br>AATCATAAATTCACTGGGTTAATAGGTATGTTATATGCGATGTCTAG<br>GTTAGGAAGAGAAGACACCATAAAAATACTCAGAGATGCGGGATAT<br>CATGTAAAAGCAAATGGAGTAGATGTAACAACACATCGTCAAGACA<br>TTAATGGAAAAGAAATGAAATTTGAAGTGTTAACATTGGCAAGCTT<br>AACAACTGAAATTCAAATCAACATTGAGATAGAATCTAGAAAATCC<br>TACAAAAAAATGCTAAAAGAAATGGGAGAGGTAGCTCCAGAATAC<br>AGGCATGACTCTCCTGATTGTGGGATGATAATATTATGTATAGCAGC<br>ATTAGTAATAACTAAATTAGCAGCAGGGGACAGATCTGGTCTTACA<br>GCCGTGATTAGGAGAGCTAATAATGTCCTAAAAAATGAAATGAAAC<br>GTTACAAAGGCTTACTACCCAAGGACATAGCCAACAGCTTCTATGA<br>AGTGTTTGAAAAACATCCCCACTTTATAGATGTTTTTGTTCATTTTGG<br>TATAGCACAATCTTCTACCAGAGGTGGCAGTAGAGTTGAAGGGATT<br>TTTGCAGGATTGTTTATGAATGCCTATGGTGCAGGGCAAGTGATGTT<br>ACGGTGGGGAGTCTTAGCAAAATCAGTTAAAAATATTATGTTAGGA<br>CATGCTAGTGTGCAAGCAGAAATGGAACAAGTTGTTGAGGTTTATG<br>AATATGCCCAAAAATTGGGTGGTGAAGCAGGATTCTACCATATATT<br>GAACAACCCAAAAGCATCATTATTATCTTTGACTCAATTTCCTCACT<br>TCTCCAGTGTAGTATTAGGCAATGCTGCTGGCCTAGGCATAATGGGA<br>GAGTACAGAGGTACACCGAGGAATCAAGATCTATATGATGCAGCAA<br>AGGCATATGCTGAACAACTCAAAGAAAATGGTGTGATTAACTACAG<br>TGTACTAGACTTGACAGCAGAAGAACTAGAGGCTATCAAACATCAG<br>CTTAATCCAAAAGATAATGATGTAGAGCTTTGAGTTAATAAAAAA

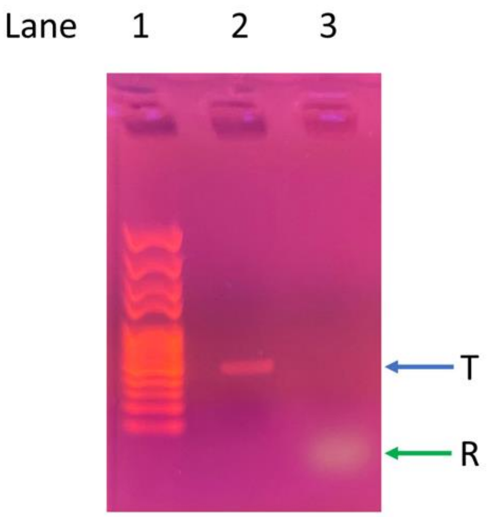

Fig. S1 Agarose gel electrophoresis was performed to verify the trans- and cis- cleavage. Lane 1: marker, Lane 2: 10 nM DNA template, Lane 3: CRISPR reaction with 10 nM DNA template. “T” indicates the location of DNA template, and “R” indicates the location of freed FAM

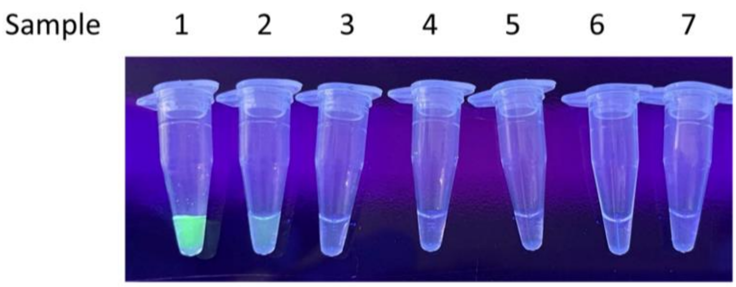

Fig. S2 Original photograph of all the tubes with CRISPR reactions. Sample 1: 10 nM DNA template; Sample 2: 1 nM DNA template; Sample 3: 100 pM DNA template, Sample 4: 10 pM DNA template, Sample 5: 1 pM DNA template, Sample 6: 100 fM DNA template; Sample 7: Negative control

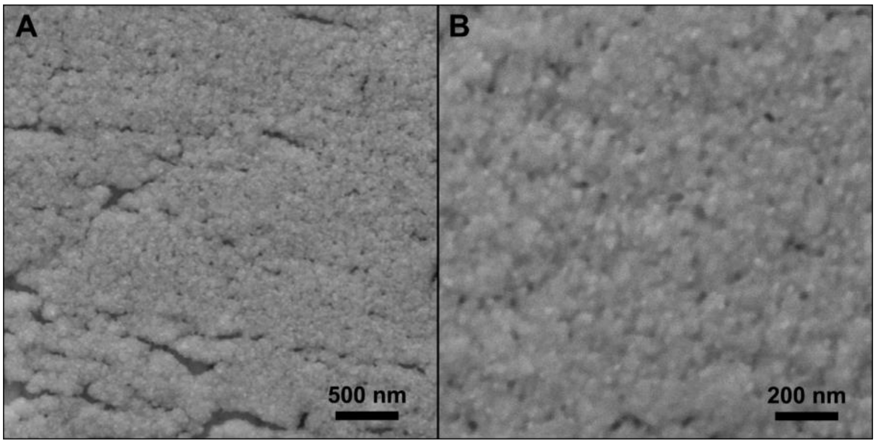

Fig. S3 SEM image of the electro-deposited AuNPs on the AuE

The SEM image was obtained from the electrode directly for accuracy with the assistance of customized AuE electrode, in which the top part of the electrode can be separated from the body of the electrode for further characterization.

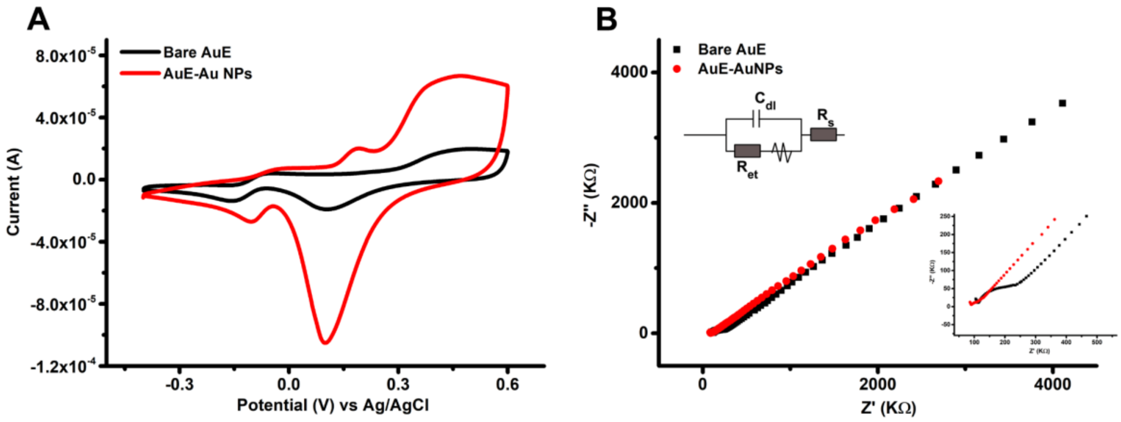

Fig. S4 CV (**A**) and EIS (**B**) curves of bare AuE and AuE-AuNPs

The CV was carried out in 0.5 M KOH with a potential range from -0.4 to 0.6 V vs Ag/AgCl at the scan rate of 100 mV/s. EIS was performed in 0.1 M KCl solution contenting 5 mM $[Fe(CN)_6]^{3-/4-}$, biased potential of 0.23 V (vs. Ag/AgCl) in the frequency range of 0.01–10^5 Hz, and 5 mV amplitude.

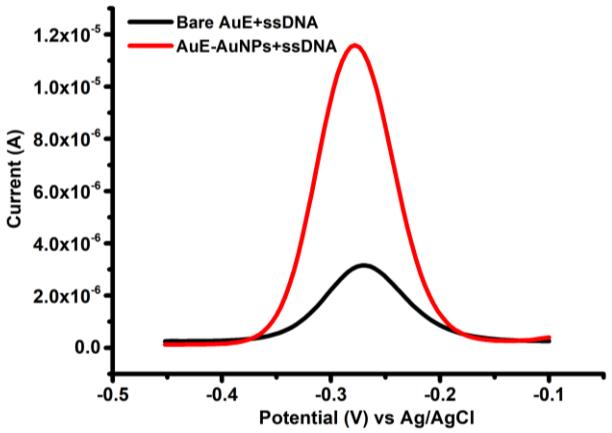

Fig. S5 The SWV of MB-ssDNA modified bare AuE and MB-ssDNA modified AuE-AuNPs

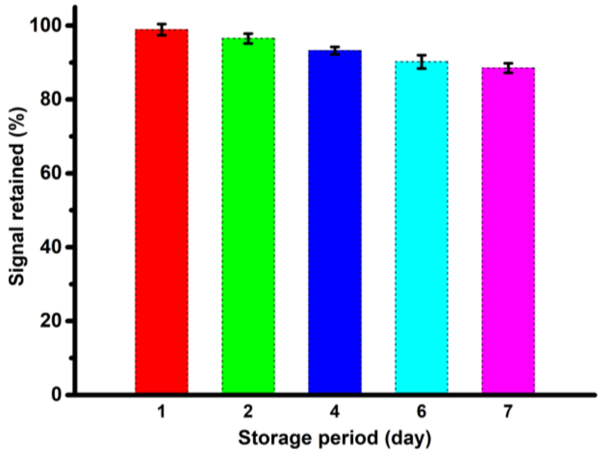

Fig. S6 Evaluation of the storage ability of the optimized ssDNA electrode surface. 100% signal was obtained through SWV test on the freshly prepared ssDNA electrode. The prepared ssDNA covered sensors were stored at 4 °C in the dark under nitrogen protection. Error bars represent standard derivation obtained in three parallel experiments

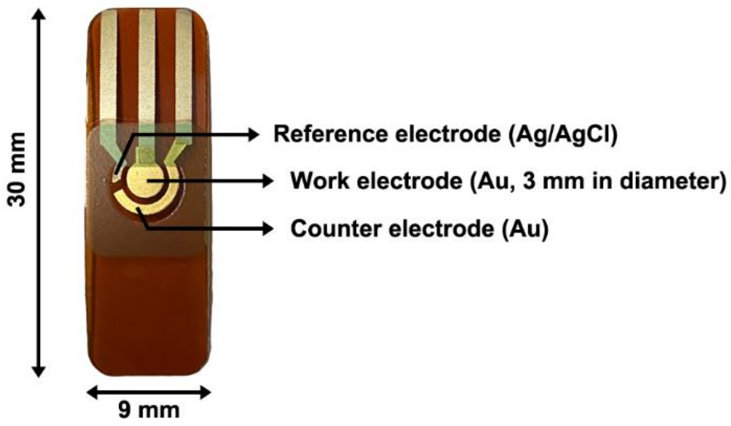

Fig. S7 Configuration of the screen-printed electrode

Supplementary Reference

[S1] M.J. Kellner, J.G. Koob, J.S. Gootenberg, O.O. Abudayyeh, F. Zhang, SHERLOCK. nucleic acid detection with CRISPR nucleases. Nat. Protoc. **14**, 2986–3012 (2019). https://doi.org/10.1038/s41596-019-0210-2
